# Supplementary material for: Inferior vagal ganglion galaninergic response to gastric ulcers
Source: PLoS One. 2020 Nov 23;15(11):e0242746. doi: 10.1371/journal.pone.0242746 (PMC7682887; doi:10.1371/journal.pone.0242746)
Supplement: S1 Table — (PDF) [file pone.0242746.s001.pdf]

Cell Body Dimensions (µm)

	Ulcer Animals		Control Animals	
Lp.	Long	Short	Long	Short
1	44,81	35,33	50,4	32,2
2	38,37	35,26	35,7	30,2
3	26,07	21,47	50	40,4
4	51,73	43,4	34	28,2
5	43,67	35,36	30,48	26,29
6	38,5	24,26	28,64	24,64
7	28,13	21,69	33,15	24,26
8	27,13	27,07	39,76	25,5
9	40,1	29,88	28,6	26,72
10	46,31	35,54	44,45	43
11	25,88	17,48	47,34	28,5
12	40,44	30,47	37,21	36,61
13	52,77	24,07	26,56	16,7
14	29,21	18,15	25,54	13,32
15	58,21	34,76	50,57	38,09
16	40,09	35,83	28,29	27,6
17	57,16	43,66	40,22	37,57
18	39,77	28,13	34,38	28,42
19	50,52	27,42	42,01	38,02
20	35,63	33,33	17,83	14,45
21	42,69	36,98	52,24	31,14
22	29,12	25,44	37,75	27,73
23	30,47	24,07	31,57	24,47
24	31,59	31,29	43,93	25,31
25	24,54	14,46	18,83	11,82
26	38,98	26,58	25,49	16,59
27	38,27	33,29	27,13	15,14
28	36,8	34,15	61,39	35,87
29	37,08	30,71	43,7	30,96
30	24,07	18,01	29,96	22,46
31	28,85	27,81	43,25	37,38
32	65,76	50,32	42,62	37,96
33	45,7	31,1	36,25	30,64
34	31,05	20,34	30,74	24,66
35	21,45	19,68	48,17	44,39
36	20,45	15,08	27,93	25,88
37	29,85	19,9	48,84	40,59
38	43,31	34,06	38,92	25,48
39	38,85	31,6	36,11	27,56
40	34,51	19,94	38,31	29,76
41	29,72	23,55		
