# Supplementary material for: Inferior vagal ganglion galaninergic response to gastric ulcers
Source: PLoS One. 2020 Nov 23;15(11):e0242746. doi: 10.1371/journal.pone.0242746 (PMC7682887; doi:10.1371/journal.pone.0242746)
Supplement: S2 Table — (PDF) [file pone.0242746.s002.pdf]

Number and percentage of PGP+/Gal+ perikarya

Control Animal	Mz 1			Mz 2			Mz 3			Mz 4			Mz 5			Mz 6		
**% GAL**		**0,443232185**			**1,009372747**			**1,035196687**			**0,31225605**			**0,485942381**			**0,784826684**	
Data		**Total**			**Total**			**Total**			**Total**			**Total**			**Total**	
	Plane	PGP	Gal	Plane	PGP	Gal	Plane	PGP	Gal	Plane	PGP	Gal	Plane	PGP	Gal	Plane	PGP	Gal
Total number of		**2933**	**13**		**2774**	**28**		**2898**	**30**		**2562**	**8**		**2881**	**14**		**3058**	**24**
Cells	1	976	4	1	834	9	1	966	10	1	737	2	1	799	5	1	870	7
	2	1142	5	2	1027	10	2	1102	11	2	1004	4	2	1108	5	2	1399	11
	3	815	4	3	913	9	3	830	9	3	821	2	3	974	4	3	789	6

Control Animal	Mz 7			Mz 8			Mz 9			Mz 10			Mz 11			Mz 12		
**% GAL**		**0,333704116**			**0,750750751**			**1,476413396**			**0,174947516**			**0,276434001**			**0,756578947**	
Data		**Total**			**Total**			**Total**			**Total**			**Total**			**Total**	
	Plane	PGP	Gal	Plane	PGP	Gal	Plane	PGP	Gal	Plane	PGP	Gal	Plane	PGP	Gal	Plane	PGP	Gal
Total number of		**2697**	**9**		**2664**	**20**		**2777**	**41**		**2858**	**5**		**2894**	**8**		**3040**	**23**
Cells	1	894	3	1	895	6	1	910	17	1	770	2	1	876	1	1	893	7
	2	1014	3	2	977	10	2	1066	10	2	1091	2	2	1078	5	2	1349	11
	3	789	3	3	792	4	3	801	14	3	997	1	3	940	2	3	798	5

Ulcer Animal	Mz 13			Mz 14			Mz 15			Mz 16			Mz 17			Mz 18		
**% GAL**		**1,570132589**			**1,766654398**			**0,610454025**			**0,523982265**			**0,813308688**			**3,42519685**	
		**Total**			**Total**			**Total**			**Total**			**Total**			**Total**	
	Plane	PGP	Gal	Plane	PGP	Gal	Plane	PGP	Gal	Plane	PGP	Gal	Plane	PGP	Gal	Plane	PGP	Gal
Total number of		**2866**	**45**		**2717**	**48**		**2621**	**16**		**2481**	**13**		**2705**	**22**		**2540**	**87**
Cells	1	959	19	1	870	17	1	896	8	1	798	3	1	859	7	1	751	24
	2	1121	14	2	1075	16	2	1004	6	2	972	5	2	1074	11	2	942	35
	3	786	12	3	772	15	3	721	2	3	711	5	3	772	4	3	847	28

Ulcer Animal	Mz 19			Mz 20			Mz 21			Mz 22			Mz 23			Mz 24		
**% GAL**		**2,181818182**			**1,654411765**			**0,893189431**			**3,746721619**			**1,077265973**			**3,072525902**	
		**Total**			**Total**			**Total**			**Total**			**Total**			**Total**	
	Plane	PGP	Gal	Plane	PGP	Gal	Plane	PGP	Gal	Plane	PGP	Gal	Plane	PGP	Gal	Plane	PGP	Gal
Total number of		**2750**	**60**		**2720**	**45**		**2687**	**24**		**2669**	**100**		**2692**	**29**		**2799**	**86**
Cells	1	948	21	1	835	14	1	846	8	1	944	29	1	854	8	1	781	24
	2	1028	24	2	1101	18	2	1059	9	2	994	33	2	1021	11	2	1142	33
	3	774	15	3	784	13	3	782	7	3	731	38	3	817	10	3	876	29
