# Supplementary material for: Inferior vagal ganglion galaninergic response to gastric ulcers
Source: PLoS One. 2020 Nov 23;15(11):e0242746. doi: 10.1371/journal.pone.0242746 (PMC7682887; doi:10.1371/journal.pone.0242746)
Supplement: S3 Table — (PDF) [file pone.0242746.s003.pdf]

Experimen 2019-01-30 16:28:03 PM CET
Instrument sds7500fast
Passive Re ROX Gal

Sample Na	Target Nai	Cт	Cт Mean	Cт SD	ΔCт Mean
Gn24A	pGAPDH	15,758952	15,697	0,097	
Gn24A	pGAPDH	15,747414			
Gn24A	pGAPDH	15,585875			
Gn24A	pGal	26,584246	26,586	0,001	10,888
Gn24A	pGal	26,586857			
Gn24A	pGal	26,586167			
GN24B	pGAPDH	15,747444	15,754	0,107	
GN24B	pGAPDH	15,863582			
GN24B	pGAPDH	15,649718			
GN24B	pGal	26,405128	26,423	0,016	10,670
GN24B	pGal	26,434175			
GN24B	pGal	26,430222			
GN25A	pGAPDH	15,519310	15,496	0,020	
GN25A	pGAPDH	15,484758			
GN25A	pGAPDH	15,483207			
GN25A	pGal	24,156254	24,114	0,038	8,619
GN25A	pGal	24,083432			
GN25A	pGal	24,103609			
GN25B	pGAPDH	15,811610	15,860	0,094	
GN25B	pGAPDH	15,800326			
GN25B	pGAPDH	15,969044			
GN25B	pGal	24,726284	24,802	0,066	8,942
GN25B	pGal	24,842023			
GN25B	pGal	24,837763			
GN26A	pGAPDH	15,386227	15,317	0,065	
GN26A	pGAPDH	15,256274			
GN26A	pGAPDH	15,309322			
GN26A	pGal	24,237127	24,195	0,037	8,878
GN26A	pGal	24,172840			
GN26A	pGal	24,174552			
GN26B	pGAPDH	15,180711	15,203	0,103	
GN26B	pGAPDH	15,112965			
GN26B	pGAPDH	15,315221			
GN26B	pGal	24,649826	24,483	0,146	9,280
GN26B	pGal	24,422879			
GN26B	pGal	24,375934			
GN27A	pGAPDH	15,219315	15,212	0,010	
GN27A	pGAPDH	15,200642			
GN27A	pGAPDH	15,215269			
GN27A	pGal	24,787868	24,791	0,002	9,579
GN27A	pGal	24,791583			
GN27A	pGal	24,792298			
GN27B	pGAPDH	15,147161	15,239	0,091	
GN27B	pGAPDH	15,328695			
GN27B	pGAPDH	15,240227			
GN27B	pGal	24,577112	24,562	0,013	9,323
GN27B	pGal	24,551908			
GN27B	pGal	24,556706			
GN28A	pGAPDH	15,301491	15,332	0,027	
GN28A	pGAPDH	15,353404			
GN28A	pGAPDH	15,342317			

GN28A	pGal	24,019470	24,021	0,002	8,689
GN28A	pGal	24,023322			
GN28A	pGal	24,021174			
GN28B	pGAPDH	15,513186	15,530	0,017	
GN28B	pGAPDH	15,531262			
GN28B	pGAPDH	15,546340			
GN28B	pGal	24,427464	24,347	0,085	8,816
GN28B	pGal	24,257715			
GN28B	pGal	24,354963			
GN29A	pGAPDH	15,028297	15,019	0,008	
GN29A	pGAPDH	15,018486			
GN29A	pGAPDH	15,011676			
GN29A	pGal	24,931931	24,950	0,018	9,930
GN29A	pGal	24,967645			
GN29A	pGal	24,949358			
GN29B	pGAPDH	15,103345	15,097	0,006	
GN29B	pGAPDH	15,090708			
GN29B	pGAPDH	15,095527			
GN29B	pGal	25,037514	25,047	0,010	9,950
GN29B	pGal	25,057833			
GN29B	pGal	25,045154			
GN30A	pGal	25,855671	25,761	0,103	10,616
GN30A	pGal	25,651927			
GN30A	pGal	25,775183			
GN30A	pGAPDH	15,318795	15,145	0,176	
GN30A	pGAPDH	14,966305			
GN30A	pGAPDH	15,150816			
GN30B	pGal	26,536530	26,398	0,131	11,255
GN30B	pGal	26,277102			
GN30B	pGal	26,380675			
GN30B	pGAPDH	15,061550	15,143	0,085	
GN30B	pGAPDH	15,231904			
GN30B	pGAPDH	15,135257			
GN31A	pGal	27,339083	27,283	0,051	12,344
GN31A	pGal	27,238947			
GN31A	pGal	27,270571			
GN31A	pGAPDH	14,961532	14,939	0,027	
GN31A	pGAPDH	14,909560			
GN31A	pGAPDH	14,944588			
GN31B	pGal	27,286618	27,200	0,088	12,096
GN31B	pGal	27,111146			
GN31B	pGal	27,202378			
GN31B	pGAPDH	15,186293	15,104	0,086	
GN31B	pGAPDH	15,014693			
GN31B	pGAPDH	15,110091			
GN32A	pGal	26,670256	26,691	0,027	11,686
GN32A	pGal	26,720704			
GN32A	pGal	26,681151			
GN32A	pGAPDH	15,105467	15,005	0,173	
GN32A	pGAPDH	14,804616			
GN32A	pGAPDH	15,103767			
GN32B	pGal	27,254152	27,040	0,222	12,058
GN32B	pGal	26,811328			
GN32B	pGal	27,055502			

GN32B	pGAPDH	15,112753	14,982	0,126	
GN32B	pGAPDH	14,861363			
GN32B	pGAPDH	14,972975			
GN33A	pGal	26,950928	26,911	0,092	11,081
GN33A	pGal	26,976183			
GN33A	pGal	26,805671			
GN33A	pGAPDH	15,780305	15,830	0,046	
GN33A	pGAPDH	15,871815			
GN33A	pGAPDH	15,838795			
GN33B	pGal	25,428102	25,348	0,100	11,255
GN33B	pGal	25,380674			
GN33B	pGal	25,235531			
GN33B	pGAPDH	14,061903	14,093	0,038	
GN33B	pGAPDH	14,135257			
GN33B	pGAPDH	14,081550			
GN34A	pGal	28,251490	28,241	0,099	12,903
GN34A	pGal	28,334707			
GN34A	pGal	28,138272			
GN34A	pGAPDH	15,369661	15,339	0,028	
GN34A	pGAPDH	15,315588			
GN34A	pGAPDH	15,330432			
GN34B	pGal	28,271048	28,200	0,081	12,246
GN34B	pGal	28,112377			
GN34B	pGal	28,216718			
GN34B	pGAPDH	15,992692	15,954	0,036	
GN34B	pGAPDH	15,921091			
GN34B	pGAPDH	15,947293			
GN35A	pGal	26,171686	26,221	0,045	11,216
GN35A	pGal	26,260115			
GN35A	pGal	26,230256			
GN35A	pGAPDH	15,103617	15,005	0,173	
GN35A	pGAPDH	14,804766			
GN35A	pGAPDH	15,105467			
GN35B	pGal	26,554227	26,575	0,022	11,353
GN35B	pGal	26,597602			
GN35B	pGal	26,574152			
GN35B	pGAPDH	15,322364	15,222	0,105	
GN35B	pGAPDH	15,231974			
GN35B	pGAPDH	15,112754			

Analysis Ty	Singleplex
Endogenou	pGAPDH
RQ Min/Ma	95.0
