# Supplementary material for: Inferior vagal ganglion galaninergic response to gastric ulcers
Source: PLoS One. 2020 Nov 23;15(11):e0242746. doi: 10.1371/journal.pone.0242746 (PMC7682887; doi:10.1371/journal.pone.0242746)
Supplement: S4 Table — (PDF) [file pone.0242746.s004.pdf]

Experimen 2019-05-30 13:51:04 PM CEST
Instrument sds7500fast
Passive Re ROX GALR1

ample Nan	arget Nam	Cт	Cт Mean	Cт SD	ΔCт Mean
GN24A	pGAPDH	17,7898	17,708	0,092	
GN24A	pGAPDH	17,7256			
GN24A	pGAPDH	17,6077			
GN24A	pGalR1	29,2939	29,533	0,208	11,826
GN24A	pGalR1	29,6644			
GN24A	pGalR2	29,6419			
GN24B	pGAPDH	17,7366	17,717	0,119	
GN24B	pGAPDH	17,5892			
GN24B	pGAPDH	17,8241			
GN24B	pGalR1	29,0296	29,050	0,026	11,333
GN24B	pGalR1	29,0397			
GN24B	pGalR2	29,0795			
GN25A	pGAPDH	17,2523	17,117	0,208	
GN25A	pGAPDH	16,8773			
GN25A	pGAPDH	17,2223			
GN25A	pGalR1	28,7296	28,734	0,018	11,616
GN25A	pGalR1	28,7536			
GN25A	pGalR2	28,7176			
GN25B	pGAPDH	17,7130	17,773	0,100	
GN25B	pGAPDH	17,7179			
GN25B	pGAPDH	17,8881			
GN25B	pGalR1	29,4573	29,673	0,187	11,900
GN25B	pGalR1	29,7727			
GN25B	pGalR2	29,7881			
GN26A	pGAPDH	17,3480	17,023	0,471	
GN26A	pGAPDH	16,4832			
GN26A	pGAPDH	17,2384			
GN26A	pGalR1	28,5189	28,598	0,165	11,575
GN26A	pGalR1	28,7882			
GN26A	pGalR2	28,4875			
GN26B	pGAPDH	17,1141	17,259	0,130	
GN26B	pGAPDH	17,2989			
GN26B	pGAPDH	17,3638			
GN26B	pGalR1	29,4859	29,299	0,171	12,040
GN26B	pGalR1	29,2587			
GN26B	pGalR2	29,1514			
GN27A	pGAPDH	16,5671	16,532	0,084	
GN27A	pGAPDH	16,5916			
GN27A	pGAPDH	16,4360			
GN27A	pGalR1	28,8436	28,713	0,122	12,182
GN27A	pGalR1	28,6933			
GN27A	pGalR2	28,6030			
GN27B	pGAPDH	17,2231	17,257	0,092	
GN27B	pGAPDH	17,1869			
GN27B	pGAPDH	17,3607			
GN27B	pGalR1	28,7199	28,751	0,027	11,494
GN27B	pGalR1	28,7718			
GN27B	pGalR2	28,7607			
GN28A	pGAPDH	17,5135	17,482	0,040	
GN28A	pGAPDH	17,4375			

GN28A	pGAPDH	17,4964			
GN28A	pGalR1	29,6239	29,623	0,001	12,141
GN28A	pGalR1	29,6223			
GN28A	pGalR2	29,6239			
GN28B	pGAPDH	17,5070	17,605	0,094	
GN28B	pGAPDH	17,6956			
GN28B	pGAPDH	17,6112			
GN28B	pGalR1	30,2641	30,258	0,007	12,653
GN28B	pGalR1	30,2592			
GN28B	pGalR2	30,2510			
GN29A	pGAPDH	17,0625	17,126	0,099	
GN29A	pGAPDH	17,0760			
GN29A	pGAPDH	17,2395			
GN29A	pGalR1	29,1783	29,106	0,076	11,980
GN29A	pGalR1	29,0268			
GN29A	pGalR2	29,1123			
GN29B	pGAPDH	17,0939	16,790	0,266	
GN29B	pGAPDH	16,5995			
GN29B	pGAPDH	16,6751			
GN29B	pGalR1	29,1185	29,148	0,026	12,358
GN29B	pGalR1	29,1686			
		29,1557			
GN30A	pGalR1	29,093309	29,328	0,266	12,286
GN30A	pGalR1	29,616554			
GN30A	pGalR1	29,274546			
GN30A	pGAPDH	16,680979	17,043	0,396	
GN30A	pGAPDH	16,980688			
GN30A	pGAPDH	17,465912			
GN30B	pGalR1	28,420267	28,689	0,266	11,189
GN30B	pGalR1	28,693869			
GN30B	pGalR1	28,952528			
GN30B	pGAPDH	17,643639	17,500	0,343	
GN30B	pGAPDH	17,748367			
GN30B	pGAPDH	17,108429			
GN31A	pGalR1	29,420479	29,605	0,169	13,015
GN31A	pGalR1	29,644241			
GN31A	pGalR1	29,750990			
GN31A	pGAPDH	16,672146	16,590	0,358	
GN31A	pGAPDH	16,900148			
GN31A	pGAPDH	16,197470			
GN31B	pGalR1	28,807268	28,860	0,143	12,383
GN31B	pGalR1	28,750782			
GN31B	pGalR1	29,022596			
GN31B	pGAPDH	16,719942	16,477	0,252	
GN31B	pGAPDH	16,494030			
GN31B	pGAPDH	16,216459			
GN32A	pGalR1	28,895060	29,123	0,305	11,899
GN32A	pGalR1	29,003262			
GN32A	pGalR1	29,469685			
GN32A	pGAPDH	17,221481	17,223	0,084	
GN32A	pGAPDH	17,308277			
GN32A	pGAPDH	17,140301			
GN32B	pGalR1	29,053450	28,980	0,074	11,802

GN32B	pGalR1	28,904989			
GN32B	pGalR1	28,981237			
GN32B	pGAPDH	16,381701	17,178	0,719	
GN32B	pGAPDH	17,780489			
GN32B	pGAPDH	17,371696			
GN33A	pGalR1	28,073209	28,295	0,207	12,371
GN33A	pGalR1	28,482554			
GN33A	pGalR1	28,328755			
GN33A	pGAPDH	15,757879	15,923	0,184	
GN33A	pGAPDH	15,891288			
GN33A	pGAPDH	16,121112			
GN33B	pGalR1	29,322267	29,523	0,188	11,289
GN33B	pGalR1	29,693869			
GN33B	pGalR1	29,552528			
GN33B	pGAPDH	18,343639	18,233	0,118	
GN33B	pGAPDH	18,248367			
GN33B	pGAPDH	18,108429			
GN34A	pGalR1	28,623499	28,774	0,422	12,184
GN34A	pGalR1	28,447241			
GN34A	pGalR1	29,250990			
GN34A	pGAPDH	16,992146	16,590	0,407	
GN34A	pGAPDH	16,600148			
GN34A	pGAPDH	16,177470			
GN34B	pGalR1	28,752269	28,526	0,435	13,049
GN34B	pGalR1	28,800782			
GN34B	pGalR1	28,024596			
GN34B	pGAPDH	15,219942	15,477	0,293	
GN34B	pGAPDH	15,414030			
GN34B	pGAPDH	15,796459			
GN35A	pGalR1	28,993660	29,147	0,160	11,723
GN35A	pGalR1	29,134262			
GN35A	pGalR1	29,313485			
GN35A	pGAPDH	17,392481	17,424	0,062	
GN35A	pGAPDH	17,495377			
GN35A	pGAPDH	17,385401			
GN35B	pGalR1	28,953460	28,880	0,064	11,958
GN35B	pGalR1	28,844989			
GN35B	pGalR1	28,841899			
GN35B	pGAPDH	16,781701	16,922	0,122	
GN35B	pGAPDH	16,992112			
GN35B	pGAPDH	16,992196			

Analysis Ty	Singleplex
Endogenou	pGAPDH
RQ Min/Ma	95.0
