# Supplementary material for: Inferior vagal ganglion galaninergic response to gastric ulcers
Source: PLoS One. 2020 Nov 23;15(11):e0242746. doi: 10.1371/journal.pone.0242746 (PMC7682887; doi:10.1371/journal.pone.0242746)
Supplement: S5 Table — (PDF) [file pone.0242746.s005.pdf]

Experime 2019-05-30 13:51:04 PM CEST
Instrume sds7500fast
Passive I ROX GalR2 Dane Zostały skompilowane z 2 p

mple Na	arget Nam	Cт	Cт Mean	Cт SD	ΔCт Mean
GN24A	pGAPDH	17,7296	17,800	0,157	
GN24A	pGAPDH	17,9798			
GN24A	pGAPDH	17,6901			
GN24A	pGalR2	32,4070	32,593	0,162	14,794
GN24A	pGalR2	32,6735			
GN24A	pGalR2	32,6999			
GN24B	pGAPDH	17,7310	17,425	0,271	
GN24B	pGAPDH	17,2155			
GN24B	pGAPDH	17,3299			
GN24B	pGalR2	33,5959	33,737	0,126	16,311
GN24B	pGalR2	33,7766			
GN24B	pGalR2	33,8372			
GN25A	pGAPDH	17,3947	17,372	0,084	
GN25A	pGAPDH	17,4416			
GN25A	pGAPDH	17,2785			
GN25A	pGalR2	30,9381	31,061	0,114	13,689
GN25A	pGalR2	31,0810			
GN25A	pGalR2	31,1639			
GN25B	pGAPDH	17,6555	17,404	0,313	
GN25B	pGAPDH	17,0543			
GN25B	pGAPDH	17,5031			
GN25B	pGalR2	33,4008	33,508	0,109	16,104
GN25B	pGalR2	33,6179			
GN25B	pGalR2	33,5050			
GN26A	pGAPDH	17,7111	17,479	0,294	
GN26A	pGAPDH	17,1486			
GN26A	pGAPDH	17,5761			
GN26A	pGalR2	31,4949	31,767	0,332	14,289
GN26A	pGalR2	32,1372			
GN26A	pGalR2	31,6695			
GN26B	pGAPDH	16,5234	16,881	0,318	
GN26B	pGAPDH	17,1312			
GN26B	pGAPDH	16,9891			
GN26B	pGalR2	32,1369	32,057	0,156	15,175
GN26B	pGalR2	31,8767			
GN26B	pGalR2	32,1565			
GN27A	pGAPDH	16,4659	16,540	0,157	
GN27A	pGAPDH	16,7198			
GN27A	pGAPDH	16,4337			
GN27A	pGalR2	31,3346	31,333	0,099	14,793
GN27A	pGalR2	31,2327			
GN27A	pGalR2	31,4308			
GN27B	pGAPDH	17,3490	17,493	0,129	
GN27B	pGAPDH	17,5328			
GN27B	pGAPDH	17,5966			
GN27B	pGalR2	32,7351	32,452	0,250	14,960
GN27B	pGalR2	32,2624			
GN27B	pGalR2	32,3596			
GN28A	pGAPDH	16,8432	16,827	0,093	
GN28A	pGAPDH	16,9116			

GN28A	pGAPDH	16,7274			
GN28A	pGalR2	32,7946	32,823	0,089	15,995
GN28A	pGalR2	32,7507			
GN28A	pGalR2	32,9227			
GN28B	pGAPDH	16,9345	17,030	0,095	
GN28B	pGAPDH	17,0296			
GN28B	pGAPDH	17,1246			
GN28B	pGalR2	34,9502	34,902	0,085	17,873
GN28B	pGalR2	34,9523			
GN28B	pGalR2	34,8045			
GN29A	pGAPDH	16,4356	16,479	0,081	
GN29A	pGAPDH	16,4288			
GN29A	pGAPDH	16,5719			
GN29A	pGalR2	33,7363	33,605	0,119	17,126
GN29A	pGalR2	33,5748			
GN29A	pGalR2	33,5032			
GN29B	pGAPDH	16,6993	16,722	0,110	
GN29B	pGAPDH	16,8420			
GN29B	pGAPDH	16,6247			
GN29B	pGalR2	35,5854	35,250	0,391	18,528
GN29B	pGalR2	34,8196			
GN29B	pGalR2	35,3438			
GN30A	pGalR2	32,2266	32,192	0,046	15,898
GN30A	pGalR2	32,2102			
GN30A	pGalR2	32,1404			
GN30A	pGAPDH	16,2652	16,295	0,042	
GN30A	pGAPDH	16,3426			
GN30A	pGAPDH	16,2763			
GN30B	pGalR2	33,7857	33,729	0,126	16,020
GN30B	pGalR2	33,5839			
GN30B	pGalR2	33,8167			
GN30B	pGAPDH	17,7991	17,709	0,080	
GN30B	pGAPDH	17,6813			
GN30B	pGAPDH	17,6463			
GN31A	pGalR2	33,5937	33,587	0,072	17,277
GN31A	pGalR2	33,6550			
GN31A	pGalR2	33,5119			
GN31A	pGAPDH	16,0007	16,309	0,495	
GN31A	pGAPDH	16,8804			
GN31A	pGAPDH	16,0471			
GN31B	pGalR2	31,9711	31,801	0,163	14,919
GN31B	pGalR2	31,7868			
GN31B	pGalR2	31,6455			
GN31B	pGAPDH	16,4205	16,882	0,476	
GN31B	pGAPDH	17,3706			
GN31B	pGAPDH	16,8547			
GN32A	pGalR2	32,5883	32,620	0,042	16,451
GN32A	pGalR2	32,6674			
GN32A	pGalR2	32,6044			
GN32A	pGAPDH	16,2699	16,169	0,180	
GN32A	pGAPDH	15,9604			
GN32A	pGAPDH	16,2756			
GN32B	pGalR2	32,3612	32,720	0,453	16,256

GN32B	pGalR2	32,5687			
GN32B	pGalR2	33,2295			
GN32B	pGAPDH	16,4900	16,463	0,039	
GN32B	pGAPDH	16,4810			
GN32B	pGAPDH	16,4192			
GN33A	pGalR2	31,2476	31,199	0,054	15,871
GN33A	pGalR2	31,2102			
GN33A	pGalR2	31,1406			
GN33A	pGAPDH	15,2652	15,328	0,057	
GN33A	pGAPDH	15,3424			
GN33A	pGAPDH	15,3763			
GN33B	pGalR2	32,7231	32,594	0,112	15,794
GN33B	pGalR2	32,5257			
GN33B	pGalR2	32,5346			
GN33B	pGAPDH	16,7991	16,800	0,127	
GN33B	pGAPDH	16,6735			
GN33B	pGAPDH	16,9275			
GN34A	pGalR2	33,6239	33,452	0,151	16,797
GN34A	pGalR2	33,3423			
GN34A	pGalR2	33,3892			
GN34A	pGAPDH	16,9276	16,655	0,266	
GN34A	pGAPDH	16,6399			
GN34A	pGAPDH	16,3972			
GN34B	pGalR2	32,7328	32,717	0,046	16,657
GN34B	pGalR2	32,7524			
GN34B	pGalR2	32,6649			
GN34B	pGAPDH	15,7853	16,060	0,310	
GN34B	pGAPDH	16,3967			
GN34B	pGAPDH	15,9976			
GN35A	pGalR2	32,1437	32,270	0,111	15,689
GN35A	pGalR2	32,3165			
GN35A	pGalR2	32,3512			
GN35A	pGAPDH	16,4997	16,581	0,131	
GN35A	pGAPDH	16,5124			
GN35A	pGAPDH	16,7324			
GN35B	pGalR2	32,1421	32,489	0,461	16,014
GN35B	pGalR2	32,3129			
GN35B	pGalR2	33,0123			
GN35B	pGAPDH	16,3999	16,475	0,065	
GN35B	pGAPDH	16,5124			
GN35B	pGAPDH	16,5120			

Analysis	Singleplex
Endogen	pGAPDH
RQ Min/I	95.0
