# Supplementary material for: Inferior vagal ganglion galaninergic response to gastric ulcers
Source: PLoS One. 2020 Nov 23;15(11):e0242746. doi: 10.1371/journal.pone.0242746 (PMC7682887; doi:10.1371/journal.pone.0242746)
Supplement: S6 Table — (PDF) [file pone.0242746.s006.pdf]

Experime 2019-05-30 13:51:04 PM CEST
Instrumer sds7500fast
Passive F ROX GalR3 Wszystkie dane z płytk

ımple Nar	Target Name	Cт	Cт Mean	Cт SD	ΔCт Mean
GN24A	pGAPDH	19,229097	19,320	0,096	
GN24A	pGAPDH	19,309810			
GN24A	pGAPDH	19,420525			
GN24A	pGalR3	30,063227	30,322	0,225	11,002
GN24A	pGalR3	30,471937			
GN24A	pGalR3	30,430649			
GN24B	pGAPDH	19,384504	19,115	0,235	
GN24B	pGAPDH	18,954609			
GN24B	pGAPDH	19,004713			
GN24B	pGalR3	30,955482	30,941	0,098	11,827
GN24B	pGalR3	31,031141	30,941		
GN24B	pGalR3	30,836800			
GN25A	pGAPDH	18,950556	18,916	0,092	
GN25A	pGAPDH	18,985963			
GN25A	pGAPDH	18,811371			
GN25A	pGalR3	29,640675	29,502	0,217	10,586
GN25A	pGalR3	29,252439			
GN25A	pGalR3	29,614204			
GN25B	pGAPDH	19,147782	19,073	0,166	
GN25B	pGAPDH	18,883365			
GN25B	pGAPDH	19,188947			
GN25B	pGalR3	30,801802	30,901	0,089	11,828
GN25B	pGalR3	30,971968			
GN25B	pGalR3	30,929137			
GN26A	pGAPDH	19,220800	18,994	0,224	
GN26A	pGAPDH	18,773916			
GN26A	pGAPDH	18,987032			
GN26A	pGalR3	29,813180	29,705	0,191	10,711
GN26A	pGalR3	29,484967			
GN26A	pGalR3	29,816757			
GN26B	pGAPDH	18,435135	18,557	0,184	
GN26B	pGAPDH	18,467225			
GN26B	pGAPDH	18,769312			
GN26B	pGalR3	29,918431	29,910	0,008	11,352
GN26B	pGalR3	29,905879			
GN26B	pGalR3	29,904283			
GN27A	pGAPDH	18,274053	18,361	0,080	
GN27A	pGAPDH	18,432469			
GN27A	pGAPDH	18,377883			
GN27A	pGalR3	30,046558	29,927	0,114	11,566
GN27A	pGalR3	29,917455			
GN27A	pGalR3	29,818354			
GN27B	pGAPDH	18,905836	19,019	0,122	
GN27B	pGAPDH	19,147607			
GN27B	pGAPDH	19,002376			
GN27B	pGalR3	30,610905	30,532	0,084	11,514
GN27B	pGalR3	30,443371			
GN27B	pGalR3	30,542839			
GN28A	pGAPDH	18,729849	18,714	0,014	
GN28A	pGAPDH	18,703192			

GN28A	pGAPDH	18,709532			
GN28A	pGalR3	30,958107	30,677	0,247	11,962
GN28A	pGalR3	30,496639			
GN28A	pGalR3	30,575173			
GN28B	pGAPDH	18,918893	18,883	0,093	
GN28B	pGAPDH	18,952778			
GN28B	pGAPDH	18,776665			
GN28B	pGalR3	32,488604	31,946	0,473	13,063
GN28B	pGalR3	31,625616			
GN28B	pGalR3	31,722628			
GN29A	pGAPDH	18,431228	18,280	0,136	
GN29A	pGAPDH	18,239754			
GN29A	pGAPDH	18,168284			
GN29A	pGalR3	31,065752	31,114	0,042	12,834
GN29A	pGalR3	31,134250			
GN29A	pGalR3	31,142750			
GN29B	pGAPDH	18,551084	18,576	0,110	
GN29B	pGAPDH	18,696355			
GN29B	pGAPDH	18,481629			
GN29B	pGalR3	31,857111	31,739	0,221	13,163
GN29B	pGalR3	31,483739			
GN29B	pGalR3	31,876732			
GN30A	pGalR3	30,36120	30,305	0,069	12,177
GN30A	pGalR3	30,22828			
GN30A	pGalR3	30,32477			
GN30A	pGAPDH	18,05794	18,128	0,064	
GN30A	pGAPDH	18,18502			
GN30A	pGAPDH	18,13974			
GN30B	pGalR3	31,58639	31,443	0,265	12,227
GN30B	pGalR3	31,60487			
GN30B	pGalR3	31,13763			
GN30B	pGAPDH	19,32707	19,216	0,099	
GN30B	pGAPDH	19,18269			
GN30B	pGAPDH	19,13747			
GN31A	pGalR3	30,85814	30,985	0,185	12,888
GN31A	pGalR3	30,89881			
GN31A	pGalR3	31,19770			
GN31A	pGAPDH	17,97191	18,097	0,229	
GN31A	pGAPDH	18,36056			
GN31A	pGAPDH	17,95728			
GN31B	pGalR3	30,37563	30,585	0,254	12,075
GN31B	pGalR3	30,57058			
GN31B	pGalR3	30,86745			
GN31B	pGAPDH	18,25742	18,509	0,345	
GN31B	pGAPDH	18,90279			
GN31B	pGAPDH	18,36699			
GN32A	pGalR3	30,40600	30,460	0,327	12,026
GN32A	pGalR3	30,16333			
GN32A	pGalR3	30,81016			
GN32A	pGAPDH	18,93702	18,434	0,461	
GN32A	pGAPDH	18,02995			
GN32A	pGAPDH	18,33599			
GN32B	pGalR3	30,95016	30,842	0,572	12,506

GN32B	pGalR3	31,35138			
GN32B	pGalR3	30,22385			
GN32B	pGAPDH	18,35450	18,336	0,024	
GN32B	pGAPDH	18,34534			
GN32B	pGAPDH	18,30850			
GN33A	pGalR2	30,61290	30,675	0,155	12,580
GN33A	pGalR2	30,85128			
GN33A	pGalR2	30,55987			
GN33A	pGAPDH	18,00134	18,094	0,084	
GN33A	pGAPDH	18,11872			
GN33A	pGAPDH	18,16324			
GN33B	pGalR2	31,21419	31,253	0,077	12,076
GN33B	pGalR2	31,34217			
GN33B	pGalR2	31,20343			
GN33B	pGAPDH	19,16577	19,178	0,059	
GN33B	pGAPDH	19,24219			
GN33B	pGAPDH	19,12517			
GN34A	pGalR2	30,99754	30,957	0,052	13,080
GN34A	pGalR2	30,97451			
GN34A	pGalR2	30,89750			
GN34A	pGAPDH	17,88121	17,877	0,120	
GN34A	pGAPDH	17,99456			
GN34A	pGAPDH	17,75418			
GN34B	pGalR2	30,44873	30,526	0,161	12,081
GN34B	pGalR2	30,41788			
GN34B	pGalR2	30,71145			
GN34B	pGAPDH	18,47412	18,445	0,146	
GN34B	pGAPDH	18,57459			
GN34B	pGAPDH	18,28749			
GN35A	pGalR2	30,25340	30,401	0,162	12,041
GN35A	pGalR2	30,37523			
GN35A	pGalR2	30,57436			
GN35A	pGAPDH	18,53422	18,360	0,217	
GN35A	pGAPDH	18,11635			
GN35A	pGAPDH	18,42879			
GN35B	pGalR2	30,85016	30,698	0,185	12,050
GN35B	pGalR2	30,75133			
GN35B	pGalR2	30,49185			
GN35B	pGAPDH	18,74570	18,648	0,086	
GN35B	pGAPDH	18,58124			
GN35B	pGAPDH	18,61750			

Analysis T	Singleplex
Endogeno	pGAPDH
RQ Min/M	95.0
